# Inkjet-Printed FASn_1–*x*_Pb_*x*_I_3_-Based Perovskite Solar Cells

**DOI:** 10.1021/acsami.4c12477

**Published:** 2024-11-07

**Authors:** Ayush Tara, Vincent Schröder, Ananta Paul, Natalia Maticiuc, Manuel F. Vasquez-Montoya, Janardan Dagar, Susheel Sharma, Rockey Gupta, Emil J. W. List-Kratochvil, Eva L. Unger, Florian Mathies

**Affiliations:** † Department of Solution Processing of Hybrid Material and Devices, Helmholtz-Zentrum Berlin, Hahn-Meitner-Platz 1, 14109 Berlin, Germany; ‡ Department of Electronics, University of Jammu, 180006 Jammu, India; § Helmholtz-Zentrum Berlin, Hahn-Meitner-Platz 1, 14109 Berlin, Germany; ∥ Department of Metallurgical Engineering and Material Science, Indian Institute of Technology Bombay, 400076 Mumbai, India; ⊥ Institut für Physik, Institut für Chemie, Humboldt-Universität zu Berlin, Zum Großen Windkanal 2, 12489 Berlin, Germany; # Department of Chemistry and Center of the Science of Materials (CSMB) Adlershof, Humboldt University of Berlin, 12489 Berlin, Germany

**Keywords:** inkjet printing, tin−lead perovskites, NIR-region solar cells, low bandgap solar cells, band gap tuning, tandem applications

## Abstract

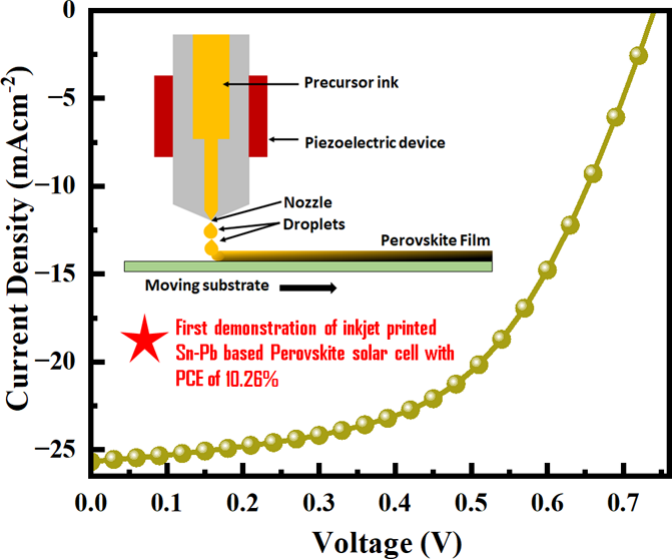

Metal halide perovskite solar cells (PSCs) have gained significant attention in thin-film photovoltaic research for their high power conversion efficiency (PCE) and facile fabrication processes. This study presents the use of inkjet printing to fabricate thin films of combinatorial mixed formamidinium tin-lead perovskites and evaluates their layer quality and device performance. Our findings demonstrate that incorporating Pb up to 50% into FASnI_3_ films enhances lattice stability. The investigation focused on optimizing the composition ratio for improved photovoltaic performance with FASn_0.5_Pb_0.5_I_3_-based PSCs achieving the highest PCE of 10.26%. Additionally, these cells exhibited an absorption spectrum extending beyond 1000 nm, corresponding to a 1.25 eV bandgap. The results suggest that inkjet printing can effectively enhance the efficiency of tin–lead-based PSCs, supporting scalability in device manufacturing.

## 1 Introduction

Perovskite material is commonly represented as ABX_3_ (with A typically representing an organic/inorganic cation, B referring to lead (Pb) or tin (Sn), and X representing a halide anion). The perovskite material has received considerable interest for its application in solar cells.^1−4^ This class of materials demonstrates advantageous optical and electrical characteristics, such as elevated absorption coefficients within the ultraviolet–visible spectrum, extended electron and hole diffusion lengths, and large grain boundaries.^5−8^ The listed attributes render these ABX_3_ perovskites highly compatible for their application in thin film solar cells as well as for photocatalytic applications, the development of light-emitting diodes, lasers, and photodetectors.^9−12^ One notable benefit of halide perovskites is their ability to undergo easy transformation into thin films through solution-processing techniques at low temperatures. Several solution-based processing techniques have been developed to prepare perovskite films of high quality, including spin coating, spray coating, slot die coating, and inkjet printing.^13−16^

Collaborative efforts from numerous research groups have led to significant advancements in the power conversion efficiency (PCE) of PSCs. In 2009, the PCE is measured at 3.8% when utilizing methylammonium (MA) lead triiodide as a sensitizer.^17^ However, with the implementation of mixed cation-based absorbers, the PCE rose above 26% in 2023.^18^ Furthermore, perovskite solar cells are raising concerns due to the escalating toxicity and environmental issues linked to the presence of Pb, with their high PCE. A key objective in the ongoing development of PSCs is to replace Pb with elements that possess lower toxicity levels. Nevertheless, the development of lead-free perovskite devices with high performance and robust stability remains a challenging task. The research teams led by Kanatzidis and Snaith have showcased lead-free Sn-based PSCs. They developed MASnI_3_-based perovskite solar cells with PCEs of 5.2 and 6.4%, respectively.^19,20^

Several efforts have been undertaken to substitute the MA cation with FA and Cesium (Cs) in pristine lead-free perovskite structures to enhance the performance of lead-free perovskite solar cells. However, PSCs based on FASnI_3_ and CsSnI_3_ have demonstrated a maximum PCE of only 14 and 11% according to studies reported to date.^21,22^ One contributing factor to the significant variations in PCE when compared with the counterpart MAPbI_3_ is the susceptibility of Sn^2+^ to air, leading to its rapid oxidation into Sn^4+^.^23^ To mitigate the issue of poor performance, implementing a controlled processing environment and effective encapsulation techniques is crucial. Additionally, pure tin perovskites are known to have poor electronic quality due to deep charge defects. However, research has shown that adding SnF_2_ can significantly reduce the density of these defects, thereby improving electronic quality.^24,25^ An alternative approach holds the potential for minimizing the use of toxic heavy metal Pb. The utilization of Sn–Pb binary perovskites in solar cells presents a significant strategy for mitigating the quantity of Pb employed. Efforts have been directed toward the development of mixed Sn–Pb perovskite devices, with a specific focus on materials based on MA and FA perovskites.^26^ Several reports have revealed that the partial substitution of lead with tin in hybrid perovskite materials results in intriguing bandgap behavior. Specifically, as the ratio of Sn/Pb is varied from 0 to 1, there is a notable reduction in the bandgap energy from 1.5 to 1.2 eV. The decrease in the bandgap leads to the expansion of the absorption spectrum toward the near-infrared region which makes them highly suitable for utilization as light absorbers in the narrow bandgap subcell of all perovskite tandem solar cells.^27,28^ To note that the inclusion of Pb^2+^ ions seems to have the capacity to stabilize Sn^2+^ ions within the perovskite structure, thereby reducing the p-doping concentration of the film in comparison to pristine Sn perovskite films.^29,30^ Nowadays, researchers are focusing on scalable techniques for the development of organometal halide perovskite solar cells, as they not only are cost-effective but also facilitate the consistent production of high-quality films necessary to achieve high-efficiency and long-term stability. Therefore, the majority of scientific investigations have been primarily directed toward the exploration of high throughput methodologies, including spray coating, blade coating, and slot die coating.^14−16^ Slot die-coated PSCs have already exhibited PCEs of over 22%.^31^ Digital inkjet printing has been studied for the fabrication of PSCs with customizable shapes and sizes. The inkjet printing process for perovskites not only offers flexibility in design but also makes it possible to precisely adjust the crystallization properties of organohalogen perovskite layers.^16^ This control is essential to achieving optimal performance in solar cells. In recent studies, inkjet-printed PSCs with a PCE of up to 21% have been successfully demonstrated.^32^ However, it is important to note that this efficiency is still lower than the reported record PCE for PSCs fabricated by using the spin coating method.

In this study, we have deposited Sn/Pb intermixed FASn_1–*x*_Pb_*x*_I_3_ (*x* = 0.25, 0.5, and 0.75)-based perovskite thin films through inkjet printing for the first time. The investigation focused on determining the ideal composition ratio to attain a favorable photovoltaic performance. The deposited FASn_1–*x*_Pb_*x*_I_3_ thin films are subjected to various characterizations followed by their implementation in solar cells. It is found that the PSCs based on FASn_0.5_Pb_0.5_I_3_ exhibited superior performance, achieving a maximum PCE of 10.26%. These findings suggest the potential for developing a Sn/Pb-based perovskite solar cell with improved efficiency through inkjet printing to work toward upscaling of the devices and for their application as top subcell in perovskite-perovskite tandem solar cells.

## 2 Experimental Section

### 2.1 Materials

The materials used to fabricate perovskite solar cells are as follows: formamidinium iodide (FAI), tin(II) iodide (SnI_2_, 99.99%), tin(II) fluoride (SnF_2_, 99%), C_60_ (99.99%), and BCP, along with dimethylformamide (DMF, 99.8%), dimethyl sulfoxide (DMSO, 99.9%), and γ-butyrolactone (GBL, 99.8%) are sourced from Sigma-Aldrich. Lead(II) iodide (PbI_2_, 99.99%) is obtained from TCI. Poly(2,3-dihydrothieno-1,4-dioxin)-poly(styrenesulfonate) (PEDOT:PSS) and silver (Ag) shots are acquired from Alfa Aesar. Indium tin oxide (ITO) substrates are sourced from Ossila.

### 2.2 Perovskite Thin Film Deposition through Inkjet Printing

The etched ITO glass substrates are ultrasonically cleaned followed by detergent, deionized water, acetone, and isopropanol for 10 min each. After blow-drying with nitrogen, the substrates are exposed to UV light for 10 min. All of the remaining steps are performed in a nitrogen-filled glovebox.

A 3 mL stock solution of FAPbI_3_ is prepared by dissolving 257.95 mg of FAI, 691.51 mg of PbI_2_ in a solvent solution of DMF, DMSO, and GBL in 4:1:1.6 v/v ratio.

Further, to prepare the 3 mL stock solution of FASnI_3_, 257.95 mg of FAI, 558.77 mg of SnI_2_, and 23.5 mg of SnF_2_ are dissolved in a solvent solution of DMF, DMSO, and GBL in 4:1:1.6 v/v ratio.

For making the three combinations of tin–lead perovskite precursor solution,

1 1 mL of FAPbI_3_ and 1 mL of FASnI_3_ is mixed to get the FASn_0.5_Pb_0.5_I_3_ precursor solution.
2 1.5 mL of FAPbI_3_ and 0.5 mL of FASnI_3_ is mixed to get the FASn_0.25_Pb_0.75_I_3_ precursor solution.
3 0.5 mL of FAPbI_3_ and 1.5 mL of FASnI_3_ is mixed to get the FASn_0.75_Pb_0.25_I_3_ precursor solution.

The solution is then filtered (0.45 μm, PTFE) before filling the inkjet printhead. A Pixdro LP50 (Süss Microtec) with a spectra SE128 printhead with a single printhead of spectra SE128 printheads (30pL droplet size) is used for perovskite thin film deposition. The ink is held at an ink-head temperature of 60 °C, and the printing is done with a printhead voltage of 80 V with a jetting frequency of 100 Hz. The best results are obtained at printing resolution of 500 dpi, quality factor 4, print speed of 100 mm/s, and drop velocity of 4m/s. After printing, the substrates are treated with gas-flow-assisted vacuum drying (GAVD)^34^ followed by thermal annealing at 100 °C for 10 min.

### 2.3 Device Fabrication

The fabrication process begins with the preparation of PEDOT:PSS films on precleaned ITO substrates using spin-coating at 6000 rpm for 45 s, followed by annealing at 120 °C for 10 min. Once the films cool to room temperature, they are transferred to a nitrogen-filled glovebox for the deposition of Sn–Pb-based perovskite films via inkjet printing. Afterward, 23 nm of C60 and 8 nm of BCP are thermally evaporated in a vacuum system at a base pressure of 10^–6^ mbar, with an evaporation rate between 0.1 and 1.0 Å/s. Finally, the 100 nm thick Ag layer is thermally evaporated through a shadow mask, where the overlap between the ITO and Ag stripes defines an active area of 0.16 cm^2^ (Figure S1).

### 2.4 Measurements and Characterization

To investigate the band edge of the perovskite absorber layer, UV–vis spectroscopy is conducted (PerkinElmer Lambda 1050 spectrophotometer). The crystal structure of the absorber is analyzed through X-ray diffraction (XRD: Bruker D8 Advanced system). Surface and cross-sectional morphology are studied by using a scanning electron microscope (SEM: Hitachi S-4100 system at 5 kV acceleration voltage). To investigate the bandgap of the perovskite films, steady-state photoluminescence was performed (PL: Edinburgh FLS 980 spectrophotometer) using a 500 nm excitation wavelength. The device’s *J*–*V* characteristics are evaluated in a nitrogen atmosphere (Oriel LCS-100 and a Keithley 2400 source-measure unit). The solar simulator is calibrated to AM1.5G using a KG3 silicon reference cell, achieving a spectral mismatch of about 0.997. To get the integrated current density, external quantum efficiency (EQE: Oriel QEPVSI-b system with a Newport 300 W xenon arc lamp) is performed. For charge state analysis of the perovskite films, X-ray photoelectron spectroscopy (XPS: an XR-50 X-ray source from SPECS with a Mg Kα anode (1253.6 eV)) is performed. To understand the band offset of the perovskite films, ultraviolet photoelectron spectroscopy (UPS: a UV HeI (21.2 eV) source at a 2.5 eV pass energy) is performed. Additional details on the XPS/UPS setup can be found in Lauermann et al.^33^

## 3 Results

**Figure 1:**
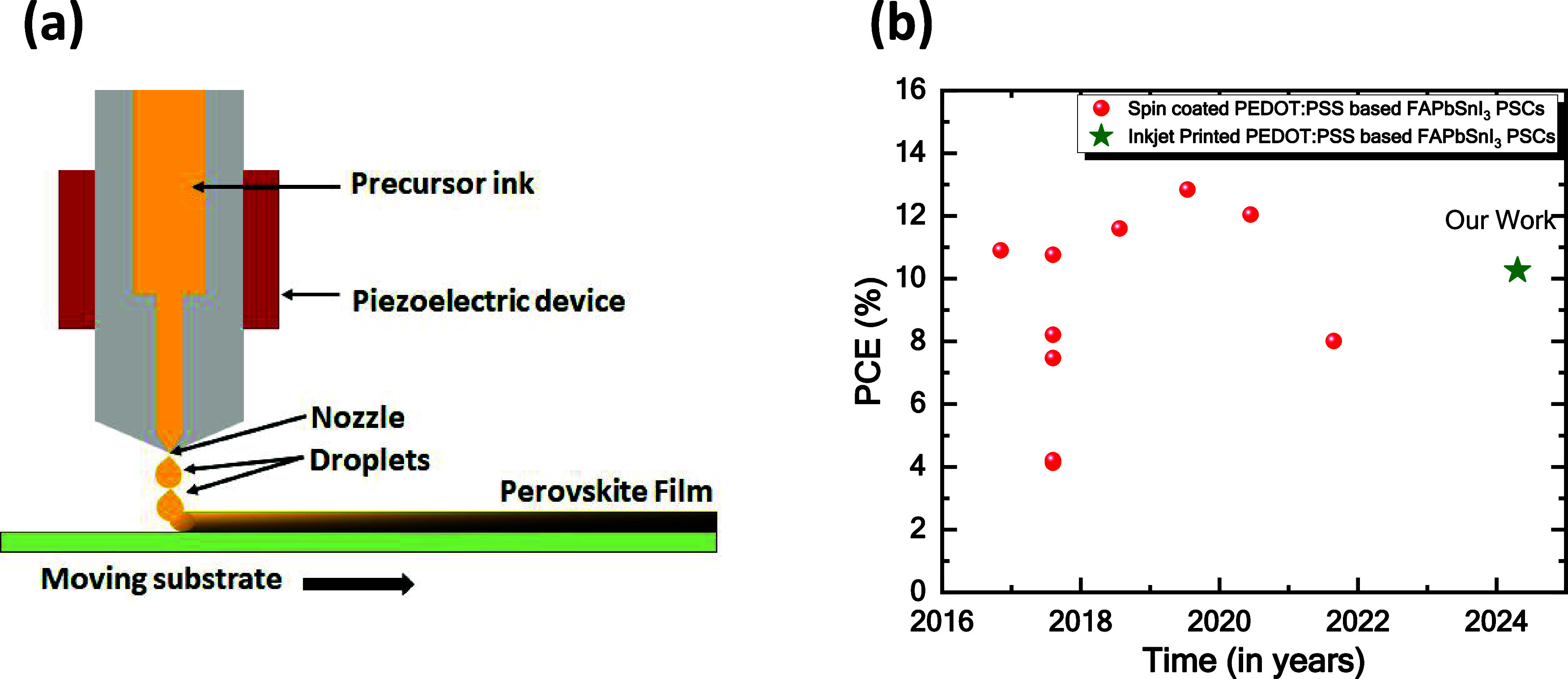
(a) Schematic illustration of inkjet printing of perovskite films and (b) comparison of the reported Sn–Pb-based perovskite solar cells with configuration glass/ITO/PEDOT:PSS/Sn–Pb perovskite/C_60_/BCP/electrode with our work.

Figure 1(a) represents a schematic diagram of the inkjet printing process. In the inkjet printing process, the droplets containing individual precursor perovskite inks (FASn_1–*x*_Pb_*x*_I_3_, *x* = 0.25, 0.5, and 0.75) are applied to the mobile substrate. In the inkjet printing technique, a specific number of ink droplets are deposited onto the substrate. The wettability of the singular droplet perovskite ink deposited on the substrate depends on the surface free energy and the surface tension of the ink. The choice of the printed image’s resolution, which is measured in dots per inch (dpi) and correlates inversely with the distance between deposited droplets, is calibrated to ensure that the deposited droplets disperse and merge upon contact, creating a cohesive wet film that is several micrometers in thickness. During the drying process of the perovskite inks, nonuniform layers can form (coffee ring effect).^34^ However, it is essential to control the crystallization process of the perovskite to maintain material quality and further optimize the performance of the resulting optoelectronic devices. Several techniques are available to facilitate the crystallization of the perovskite, including annealing at various temperatures, vacuum drying, and gas-flow-assisted drying, which can aid in the drying process. Figure 1(b) represents an overall picture of the spin-coated vs inkjet printing-based perovskite solar cells over the years from 2016 to 2024 (details of the comparison are mentioned in Table S1 and Figure S2). Here, we have deposited FASn_1–*x*_Pb_*x*_I_3_ (*x* = 0.25, 0.5, and 0.75) perovskite ink on clean indium tin oxide (ITO) substrates. After printing the perovskite ink, the perovskite-coated substrates are treated with N_2_ gas-flow-assisted vacuum drying, followed by thermal annealing at 100 °C for 10 min.^35^

**Figure 2:**
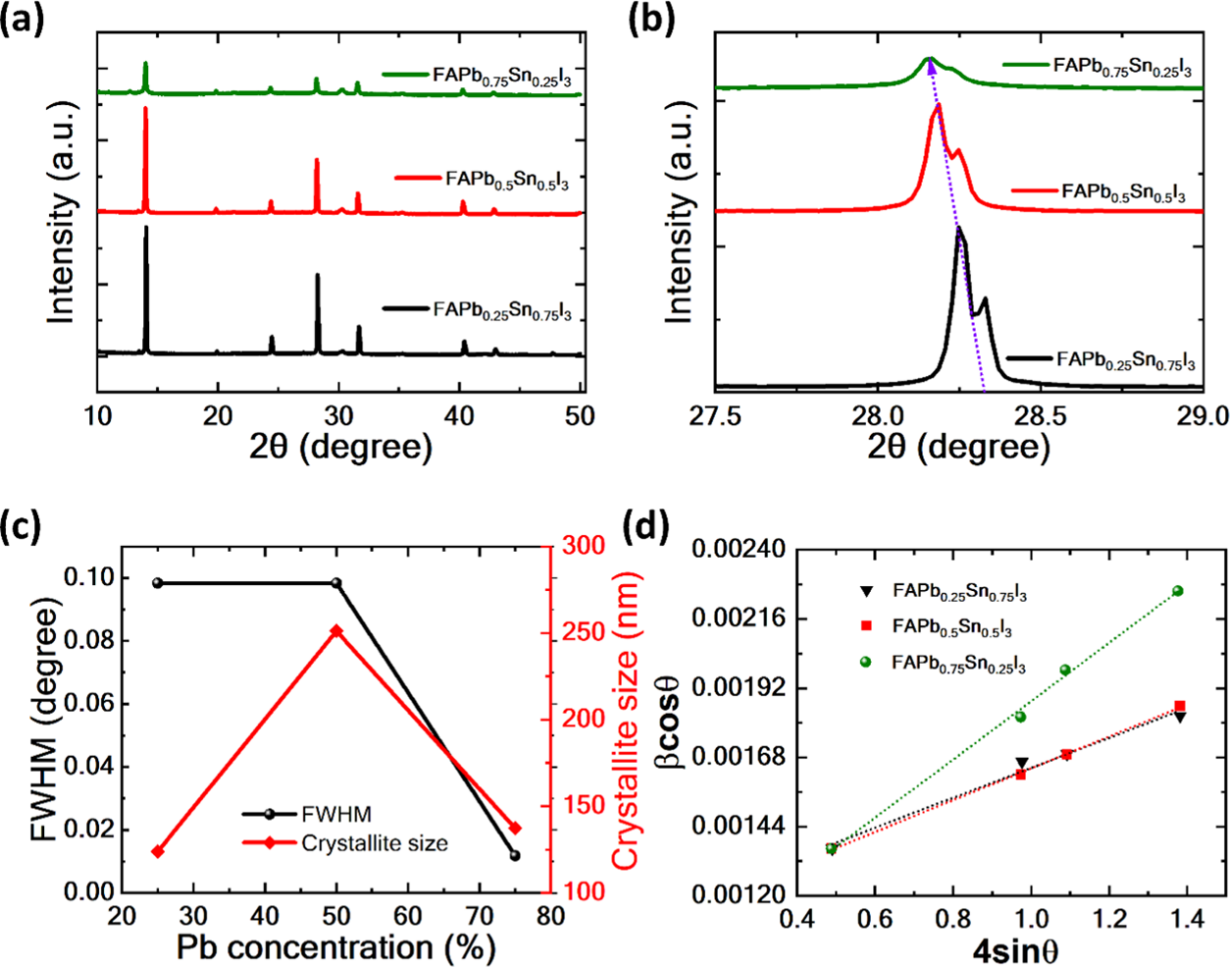
(a, b) XRD spectra and enlargement of the ∼28° peak of the FAPb_1–*x*_Sn_*x*_I_3_ perovskite films. (c) Calculated FWHM and crystallite size of inkjet-printed perovskite films. (d) Williamson–Hall plots for each sample.

Figure 2(a) reveals the XRD patterns of FASn_1–*x*_Pb_*x*_I_3_ (*x* = 0.25, 0.5, and 0.75) perovskite-based films on the glass/ITO substrate. The observed series of diffraction peaks at 14.5, 20.4, 24.9, 28.7, 32.1, 40.8, and 43.4° correspond to the standard diffraction peaks of FASn_1–*x*_Pb_*x*_I_3_ perovskite.^36^ The XRD peaks observed at 14.5 and 28.7° confirm the characteristic peaks of the perovskite.^37^ The incorporation of Pb at concentrations of 25, 50, and 75% results in a decrease in the diffraction peak intensity. It is observed that the peak intensity is highest at a 25% Pb concentration and diminishes with further increases in the Pb content. Notably, perovskite films with 25 and 50% Pb concentrations demonstrate higher crystallinity compared to a 75% Pb concentration. This suggests that a higher concentration of Pb may induce lattice distortion or disorder, contributing to the poorer crystallinity of the film and a higher trap density.^38^ For a better understanding of the XRD pattern, we enlarge the diffraction peak at ∼28° shown in Figure 2(b). The diffraction peak observed at ∼28° slightly blue-shifted to a lower 2θ angle. This result also confirms that the lattice parameter increases with increasing the Pb concentration. The variations in lattice parameters with different Pb concentrations are detailed in Table S2.

Figure 2(c) illustrates the variations in the full width at half-maximum (FWHM) and crystallite size for Pb-doped perovskite films with Pb concentrations of 25, 50, and 75%. Interestingly, the introduction of Pb into the perovskite matrix shows no significant change in FWHM up to 50%, but beyond this point, a decrease in FWHM is observed. On the other hand, the crystallite size of the FASn_1–*x*_Pb*x*I_3_ perovskite films shows an increasing trend upto 50% Pb incorporation and after that a decrease trend is observed beyond 50% of Pb incorporation.

Additionally, we have chosen the four diffraction peaks including ∼14, ∼28, ∼31, and 40° to calculate the presence of lattice strain in perovskite films using the Williamson–Hall (W–H) method.^39,40^ This approach takes into account two factors to analyze the broadening of diffraction peaks: crystallite-size-induced broadening (β_l_)(1) and strain-induced broadening (β_e_)(2).^41^
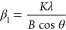
1where *B* is the crystallite size, β is the fwhm of the diffraction peak, and λ is the X-ray wavelength. The strain-induced broadening β_e_ (2) is calculated from
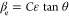
2where ε represents the strain, and *C* is a constant (typically around 4).^41^ In the presence of both crystallite-size and strain-induced broadening, their combined effects should be determined by convolution. As a first approximation, we can add up the two contributions
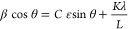
3

The corresponding W–H plots with different Pb concentrations are shown in Figure 2(d). From the positive slopes of the fitting curves, we conclude a lattice expansion in all perovskite samples. The slopes of the Williamson–Hall (W–H) plots for films with varying Pb concentrations are 0.000519 ± 0.00025, 0.00101 ± 0.00015, and 0.000552 ± 0.00035, respectively, indicating differences in the degree of lattice strain. This result indicates that perovskite films containing 50% Pb exhibit significant lattice strain. In theory, the relaxation of this strain helps reduce nonradiative recombination, thereby improving the efficiency.

**Figure 3:**
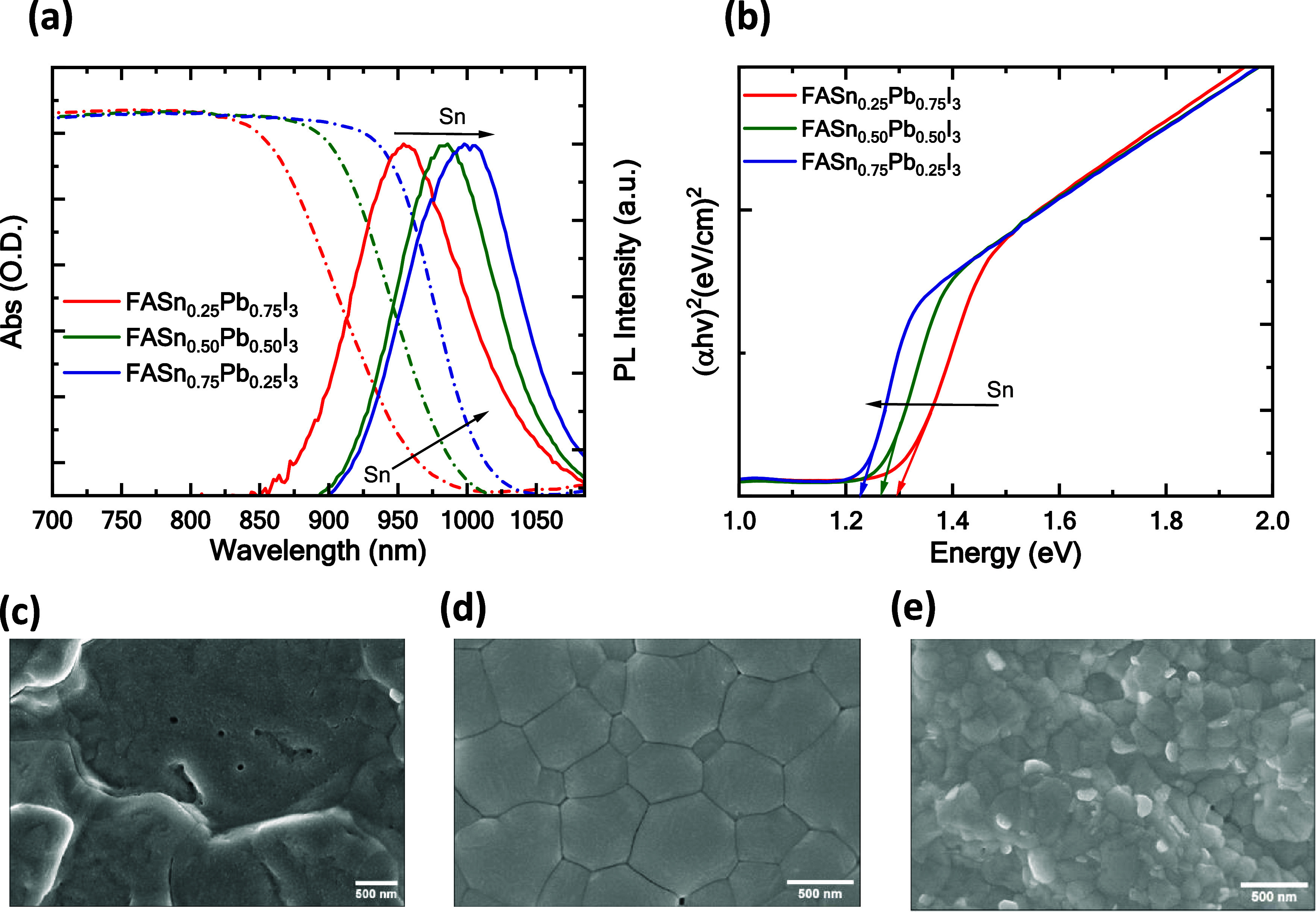
(a) Absorbance and corresponding PL spectra, (b) Tauc plot of FASn_1–*x*_Pb_*x*_I_3_ thin films, (c) SEM image of FASn_0.25_Pb_0.75_I_3_ film, (d) SEM image of FASn_0.5_Pb_0.5_I_3_ film, and (e) SEM image of FASn_0.75_Pb_0.25_I_3_ film.

Figure 3(a) depicts the absorbance and corresponding photoluminescence (PL) intensity of the FASn_1–*x*_Pb_*x*_I_3_ perovskite films on the glass/ITO substrates. A redshift in the absorption spectrum is evident and corroborated by the PL spectroscopy data. The incorporation of Pb atoms into the perovskite matrix results in a blue shift of the absorption edge by 50 nm. Additionally, Figure 3(a) illustrates that the FASn_0.75_Pb_0.25_I_3_, FASn_0.5_Pb_0.5_I_3_, and FASn_0.25_Pb_0.75_I_3_ samples shows PL peaks at 1002.28, 984.38, and 955.52 nm, respectively. Notably, the stoichiometric mixing of divalent metal iodides of Sn and Pb with FAI enables the synthesis of the FASn_1–*x*_Pb_*x*_I_3_ perovskite solution, offering a tunable bandgap ranging from 1.23 to 1.29 eV. The bandgap of the FASn_1–x_Pb_*x*_I_3_ perovskite layers has been determined using Tauc plots, considering various Pb compositions (*x* = 0.25, 0.50, and 0.75).^42^

Our measurements indicate that the bandgaps of the perovskite layers are 1.23 1.25, and 1.29 eV for *x* = 0.25, 0.5, and 0.75, respectively. These values are determined from the (α*h*ν)^2^ ∼ *h*ν relationship, as shown in Figure 3(b). To ensure adequate surface coverage of the perovskite films, scanning electron microscopy (SEM) is performed. Figure 3(c–e) represent the SEM images of Pb-doped FASn_1–*x*_Pb_*x*_I_3_ (*x* = 0.25, 0.5, 0.75) perovskite-based films on glass/ITO/PEDOT:PSS substrates Notably, the perovskite film with a 50% Pb concentration exhibits superior film quality, characterized by uniform coverage and high crystallinity, compared to films with 25 and 75% Pb concentrations. Films with 25% Pb concentration show improper grain formation and numerous pinholes, whereas films with 75% Pb concentration shows uneven grain formation, resulting in subpar film quality. It is observed that perovskite films doped with 50% Pb exhibit an average grain size of approximately 680 nm, with uniform coverage as determined through ImageJ software analysis.^43^ The results indicate that a 50% Pb concentration has the potential to improve the crystallinity of the perovskite film when grown on PEDOT:PSS films coated on ITO substrates.

**Figure 4:**
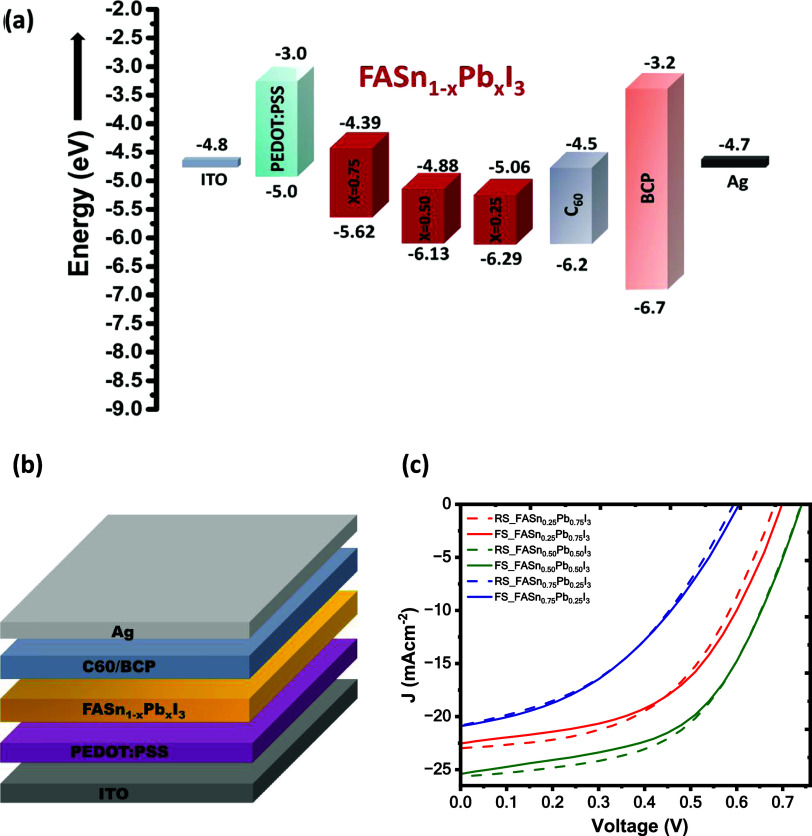
(a) Corresponding band diagram calculated from UPS analysis. (b) Device architecture. (c) *J–V* curves of FASn_1–*x*_Pb_*x*_I_3_-based PSCs.

To quantify the charge states of FASn_1–*x*_Pb_*x*_I_3_ films coated on ITO substrates X-ray photoelectron spectroscopy (XPS) analysis is performed (Figure S3). XPS confirms the presence of core energy levels I 3d, Pb 4f, and Sn 3d within this perovskite compound. For identification of the energy-level alignment of FASn_1–*x*_Pb_*x*_I_3_ films, ultraviolet photoelectron spectroscopy (UPS) measurements are performed. Figure S4 displays the UPS spectra on the valence-band regions (*E*_onset_) and secondary electron cutoffs (*E*_cutoff_) of FASn_1–*x*_Pb_*x*_I_3_ (*x* = 0.25, 0.5, and 0.75) films on ITO glass substrates. As illustrated in Figure S4, the *E*_onset_ values are determined as 1.62, 1.97, and 2.14 eV, and the *E*_cutoff_ values are 17.22, 17.05, and 17.07 eV for FASn_0.25_Pb_0.75_I_3_, FASn_0.5_Pb_0.5_I_3_, and FASn_0.75_Pb_0.25_I_3_ perovskite films, respectively. The valence-band maximum (VBM) of Sn–Pb perovskite films can be calculated using the following equation
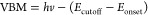
4where He(I) source incident photon energy *h*ν = 21.22 eV of UPS measurement systems. The calculated valence band maxima (VBMs) are determined to be −5.625, −6.137, and −6.295 eV for FASn_0.25_Pb_0.75_I_3_, FASn_0.5_Pb_0.5_I_3_, and FASn_0.75_Pb_0.25_I_3_ perovskite films, respectively. However, we observed that the VBM decreases from −5.625 to −6.295 eV as the Pb concentration decreases from x = 0.75 to 0.25, consistent with previous findings.^44^ The conduction-band minimums (CBM) of FASn_0.25_Pb_0.75_I_3_, FASn_0.5_Pb_0.5_I_3_, and FASn_0.75_Pb_0.25_I_3_ perovskite films are derived from their optical bandgap and calculated CBMs, as −4.39, −4.88, and −5.06 eV, respectively. Figure 4(a) demonstrates this band diagram of FASn_1–*x*_Pb_*x*_I_3_ (*x* = 0.25, 0.5, and 0.75) with the respective electron transport layer (ETL) and hole transport layer (HTL) used in the device, which is based on the UPS spectra as mentioned in Figure S4.

Figure 4(b) illustrates the schematic of the mixed Pb–Sn-based FASn_1–*x*_Pb_*x*_I_3_ perovskite solar cell, featuring a structure of Glass/ITO/PEDOT:PSS/Sn–Pb Perovskite/C_60_/BCP/Ag. Complementary cross-sectional SEM images of the FASn_0.5_Pb_0.5_I_3_ perovskite thin film and the corresponding device, shown in Figure S5, further confirm the crystalline structure of the perovskite layer. Figure 4(c) represents the *J*–*V* characteristics for the FASn_1–*x*_Pb_*x*_I_3_-based devices with different compositions. However, the FASn_0.5_Pb_0.5_I_3_ composition demonstrated superior device performance compared to that of the FASn_0.75_Pb_0.25_I_3_ and FASn_0.25_Pb_0.75_I_3_-based PSCs. The hybrid FASn_0.5_Pb_0.5_I_3_ film shows the formation of smaller crystallite domains, indicating a more favorable crystal growth of the mixed metal perovskite, leading to a more homogeneous film formation.^45,46^ Detailed performance data of the champion devices are listed in Table 1. The top-performing FASn_0.5_Pb_0.5_I_3_-based solar cell achieved an open-circuit voltage (*V*_OC_) of 0.74 V, a short-circuit current density (*J*_SC_) of 25.66 mA cm^–2^, a fill factor (FF) of 54.06%, and a PCE of 10.26% under reverse scan conditions and 1-sun illumination conditions. PCE statistics for the FASn_1–*x*_Pb_*x*_I_3_-based solar cell devices, where *x* = 0.75, 0.5, and 0.25 along with standard deviation is illustrated in Figure S6. The EQE of the FASn_0.5_Pb_0.5_I_3_-based device ranges over the entire visible spectrum up to 1000 nm with a broad absorption maximum from 500 to 800 nm. The integrated *J*_SC_ for the FASn_0.5_Pb_0.5_I_3_-based device, calculated from EQE curves, reaches 23.22 mA cm^–2^, which almost matches the measured *J*_SC_ values from *J–V* measurements (Figure S7). The obtained fill factor (FF) is 54.06% for FASn_0.5_Pb_0.5_I_3_-based device is notably lower compared to reported values.^47^

**Table 1:** Photovoltaic Parameters of the FASn_1–x_Pb_*x*_I_3_ PSCs (Active Area of the Device is 0.16 cm^2^)

		*V*_OC_ (V)	*J*_SC_ (mA cm^–2^)	FF (%)	PCE (%)
FASn_0.25_Pb_0.75_I_3_-based PSC	forward	0.69	22.52	51.86	8.05
	reverse	0.68	22.97	51.42	8.03
FASn_0.50_Pb_0.50_I_3_-based PSC	forward	0.74	25.33	53.67	10.06
	reverse	0.74	25.66	54.06	10.26
FASn_0.75_Pb_0.25_I_3_-based PSC	forward	0.60	20.89	41.13	5.19
	reverse	0.59	20.84	42.04	5.20

## 4 Discussion

The study explores the benefits of inkjet printing for creating Sn–Pb-based perovskite solar cells, specifically the FASn_1–*x*_Pb_*x*_I_3_ compositions. The inkjet process enables precise droplet deposition, with efficiency dependent on the wettability influenced by surface tension and energy. Proper calibration ensures uniform film thickness, while control over crystallization is essential to avoid defects like the “coffee ring effect”. XRD analyses confirm successful perovskite formation, showing that higher Pb concentrations can lead to reduced crystallinity. Optical properties indicate a redshift in absorption with tunable bandgaps between 1.23 and 1.29 eV. SEM reveals that the optimal 50% Pb concentration produces the highest-quality films, while lower and higher concentrations display grain formation issues. XPS and UPS provide insights into energy-level alignment, supporting an effective charge transport mechanism. The FASn_0.5_Pb_0.5_I_3_ solar cell demonstrates superior performance with a short-circuit current density of 25.66 mA/cm^2^ and a power conversion efficiency of 10.26%. Overall, the findings highlight the potential of inkjet-printed Sn–Pb-based perovskite solar cells for future advancements in solar technology.

## 5 Conclusions

To summarize, we have demonstrated the fabrication of low-bandgap FASn_1–*x*_Pb_*x*_I_3_ (*x* = 0.25, 0.50, and 0.75) perovskite solar cells using inkjet printing. Notably, the device based on the FASn_0.5_Pb_0.5_I_3_ perovskite achieved a remarkable PCE of 10.26%. To our knowledge, this result represents the highest reported efficiency for mixed Sn–Pb-based perovskite solar cells produced through inkjet printing to date. The FASn_1–*x*_Pb_*x*_I_3_ perovskite shows a widened light absorption range into the near-infrared, as confirmed by optical measurements and EQE spectra. The FASn_0.5_Pb_0.5_I_3_ perovskite solar cell achieved a high short-circuit photocurrent density of 25.66 mA/cm^2^ under 1-Sun illumination. These results highlight an effective strategy for achieving efficient low-bandgap perovskite solar cells, paving the way for further advancements in the field.

## Supporting Information Available

Comparison of FASn_1–*x*_Pb_*x*_I_3_ PSCs based on PEDOT:PSS HTL; XRD, XPS, UPS, cross-section SEM, PSCs device statistics, and IPCE (PDF)
