# Supplementary material for: Inkjet-Printed FASn1–xPbxI3-Based Perovskite Solar Cells
Source: ACS Appl Mater Interfaces. 2024 Nov 7;16(46):63520–7. doi: 10.1021/acsami.4c12477 (PMC11583116; doi:10.1021/acsami.4c12477)
Supplement: Supplementary file 1 — am4c12477_si_001.pdf [file am4c12477_si_001.pdf]

Supporting Information

Inkjet-printed $FASn_{1-x}Pb_xI_3$-based perovskite solar cells

Ayush Tara[1,2], Vincent Schröder[3], Ananta Paul[4], Natalia Maticiuc[1], Manuel F. Vasquez-Montoya[1], Janardan Dagar[1], Susheel Sharma[2], Rockey Gupta[2], Emil List-Kratochvil[3,5,6], Eva L. Unger[1,6] and Florian Mathies[1]*

[1]*Department of solution processing of hybrid material and devices, Helmholtz-Zentrum Berlin, Hahn-Meitner-Platz 1, 14109 Berlin, Germany*

[2]*Department of Electronics, University of Jammu, 180006 Jammu, India*

[3]*Helmholtz-Zentrum Berlin, Hahn-Meitner-Platz 1, 14109 Berlin, Germany*

[4]*Department of Metallurgical Engineering and Material Science, Indian Institute of Technology Bombay, 400076 Mumbai, India*

[5]*Institut für Physik, Institut für Chemie, Humboldt-Universität zu Berlin, Zum Großen Windkanal 2, 12489 Berlin, Germany*

[6]*Department of Chemistry and Center of the Science of Materials (CSMB) Adlershof, Humboldt University of Berlin, 12489 Berlin, Germany*

*Email: florian.mathies@helmholtz-berlin.de

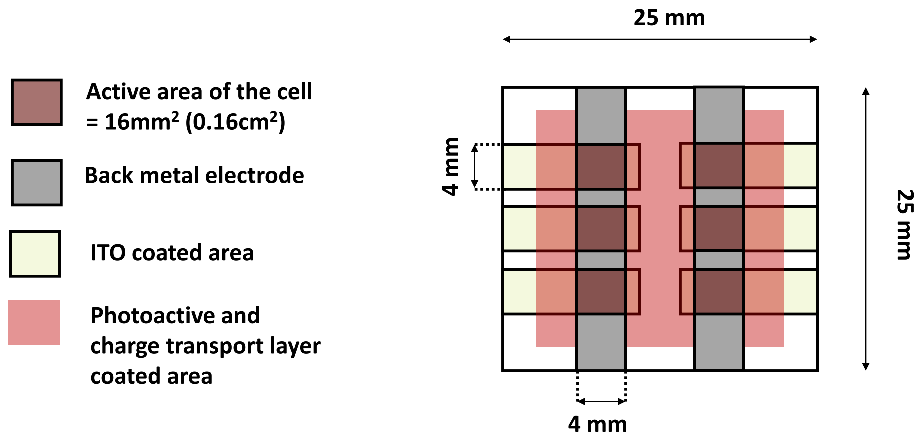

Figure S1: Illustration of the ITO substrate and various film coated areas along with active area of the perovskite solar cell device

Table S1: Comparison of $FASn_{1-x}Pb_xI_3$ PSCs based on PEDOT:PSS HTL (extracted from https://www.perovskitedatabase.com/)[1]

S. No.	Device Structures	PCE with J-V measurement conditions	Ref.
1.	ITO/PEDOT:PSS/$FAPb_{0.5}Sn_{0.5}I_3$/PCBM/Ag	10.76 %, measured under ambient environment	[2]
2.	ITO/PEDOT:PSS/$FAPb_{0.7}Sn_{0.3}I_3$/PCBM/BCP/Ag	12.84%, measured in N_2 filled glove box	[3]
3.	ITO/PEDOT:PSS/$FAPb_{0.5}Sn_{0.5}$I3/C_{60}/BCP/Al	11.60%, measured in N_2 filled glove box	[4]
4.	ITO/PEDOT:PSS/$FAPb_{0.5}Sn_{0.5}I_3$/C_{60}/BCP/Ag	10.9%, measured under ambient environment	[5]
5.	ITO/PEDOT:PSS/$FAPb_{0.5}Sn_{0.5}I_3$/C_{60}/BCP/Al	8.01%, measured in N_2 filled glove box	[6]
6.	ITO/PEDOT:PSS/$FAPb_{0.5}Sn_{0.5}I_3$/C_{60}/BCP/Ag	12.04%, measured in N_2 filled glove box	[7]
7.	ITO/PEDOT:PSS/$FAPb_{0.5}Sn_{0.5}I_3$/C_{60}/BCP/Ag	10.26%, measured in N_2 filled glove box	This Work

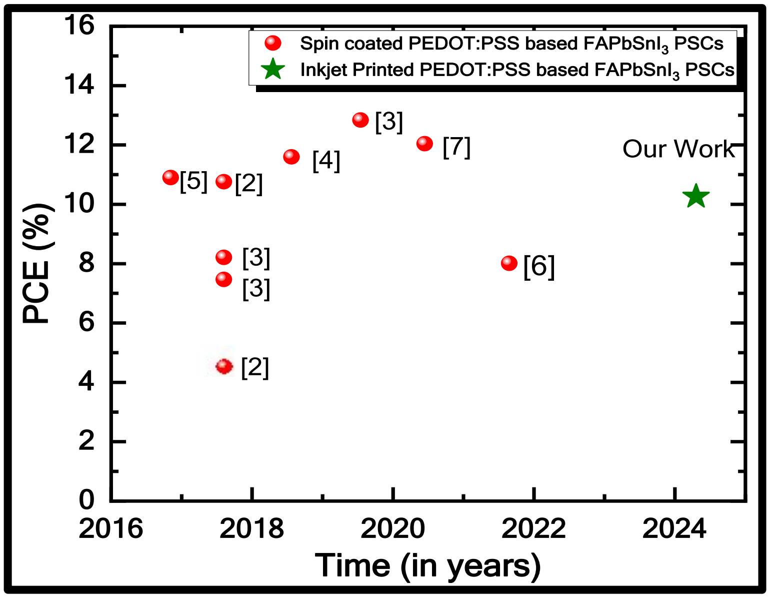

Figure S2: Summary of the reported Sn-Pb based perovskite solar cells with configuration ***Glass/ITO/PEDOT:PSS/Sn-Pb Perovskite/ETL/Electrode***

NOTE:1

XRD analysis:

Bragg's diffraction condition is

$$2dsin\theta = n\lambda \text{ .... } (1)$$

Where, d is lattice spacing, θ Bragg's diffraction angle and λ is wavelength of the Xray.

Now we also know that the lattice spacing

$$d = \frac{a}{\sqrt{h^2+k^2+l^2}} \text{ .... } (2)$$

Where, a is the lattice parameter and h, k, l are miller indices.

Now from equation (1) and (2) gives

$$a = \frac{n\lambda\sqrt{h^2+k^2+l^2}}{2dsin\theta} \text{........... } (3)$$

Table S2: Variation of lattice parameters with increasing Pb concentration

Pb concentration	2θ (degree)	d (Å)	hkl	Lattice parameter (Å)	Average lattice parameter (Å)
75 % pb	14.0026	6.3170	001	6.3170	6.3256
	28.1613	3.1649	002	6.3298	
	31.5664	2.8309	012	6.3300	
50%	14.0084	6.3144	001	6.3144	6.3200
	28.1850	3.1623	002	6.3246	
	31.6115	2.8269	012	6.3211	
25%	14.0535	6.2943	001	6.2943	6.3030
	28.2547	3.1547	002	6.3094	

	31.6919	2.8199	012	6.3054	

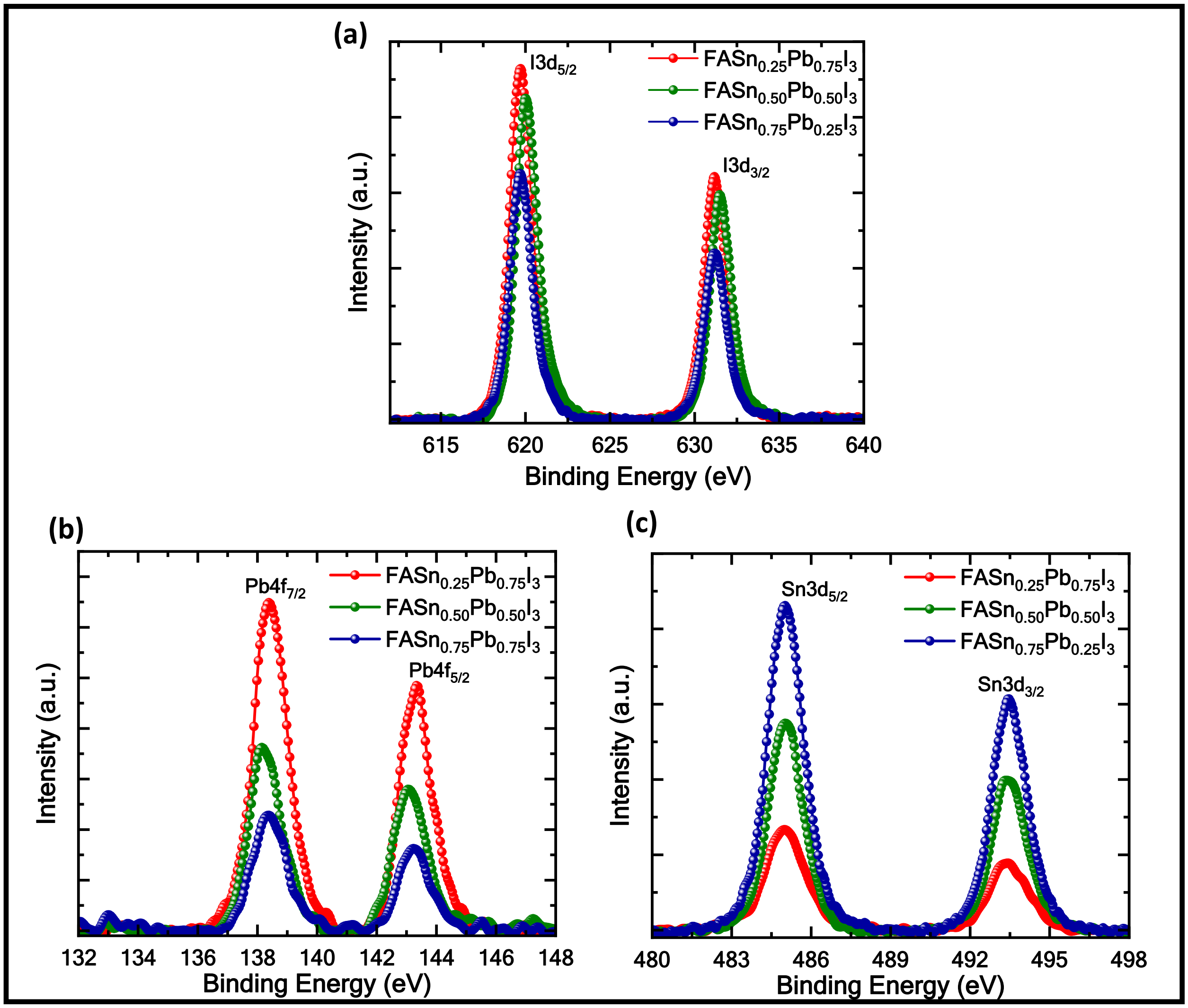

Figure S3: XPS spectra of (a) I3d, (b) Pb4f and (c) Sn3d for three different $FASn_{1-x}Pb_xI_3$ films coated on ITO substrates, where x= 0.75, 0.5, 0.25

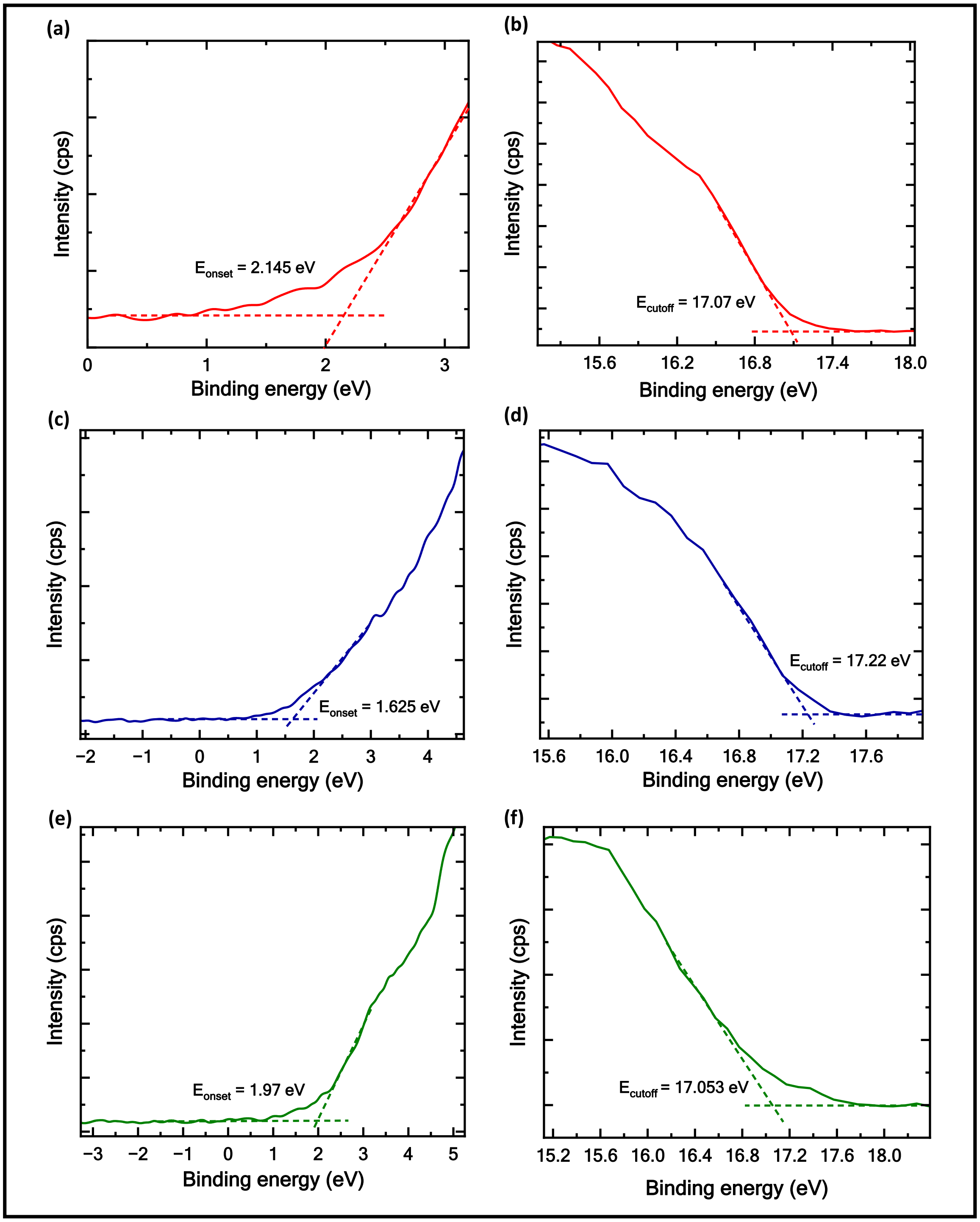

Figure S4: UPS spectra for (a) valence band regions (E_{onset}) and (b) secondary electron cutoffs (E_{cutoff}) for $FASn_{0.25}Pb_{0.75}I_3$ perovskite film; (c) valence band regions (E_{onset}) and (d) secondary electron cutoffs (E_{cutoff}) for $FASn_{0.5}Pb_{0.5}I_3$ perovskite film and (e) valence band regions (E_{onset}) and (f) secondary electron cutoffs (E_{cutoff}) for $FASn_{0.75}Pb_{0.25}I_3$ perovskite film coated on ITO substrates

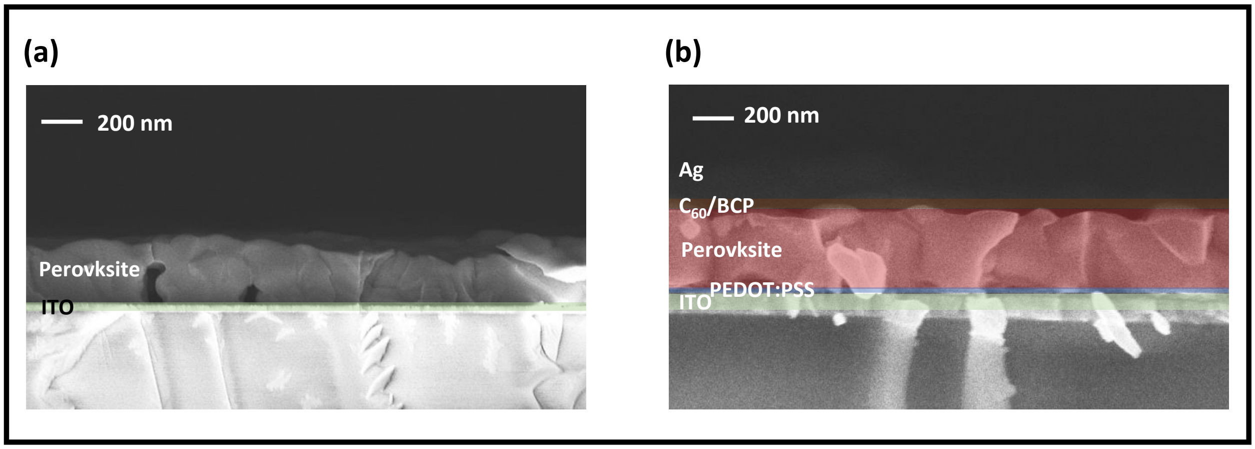

Figure S5: (a) Cross-section SEM images of $FASn_{0.5}Pb_{0.5}I_3$ perovskite thin film and (b) $FASn_{0.5}Pb_{0.5}I_3$ based solar cell device

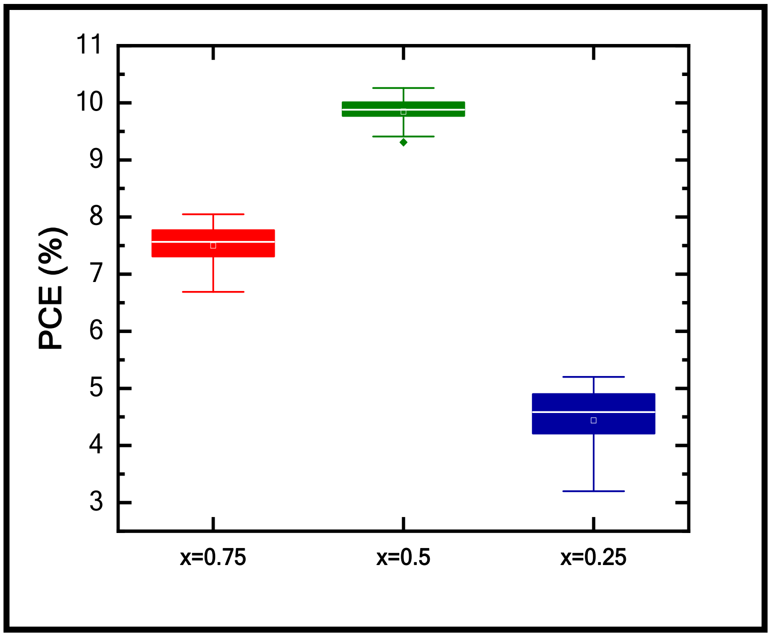

Figure S6: PCE statistics for the $FASn_{1-x}Pb_xI_3$ based solar cell devices, where x= 0.75, 0.5, 0.25 (Boxes indicate the standard deviation)

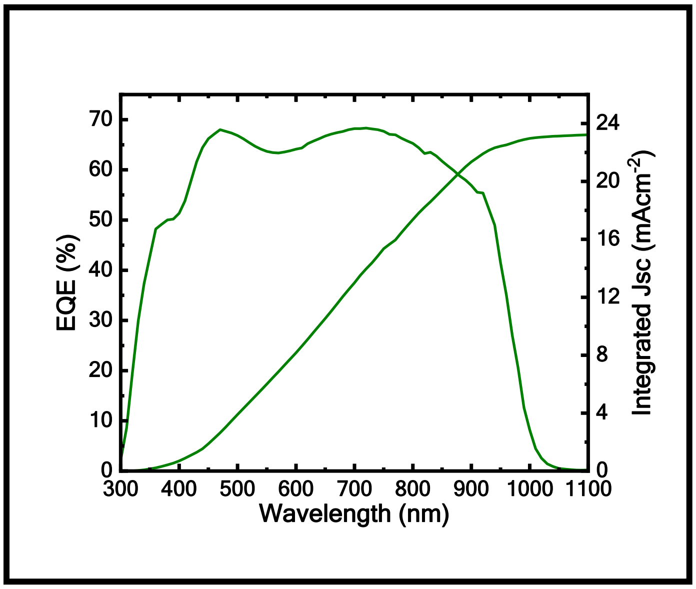

Figure S7: IPCE of $FASn_{0.5}Pb_{0.5}I_3$ perovskite solar cell device

References:

[1] Jacobsson T. J.; Hultqvist A.; García-Fernández A.; Anand A.; Al-Ashouri A.; Hagfeldt A.; Crovetto A. et al. An open-access database and analysis tool for perovskite solar cells based on the FAIR data principles. *Nature Energy* **2022**, 7 (1), 107-115, https://doi.org/ 10.1038/s41560-021-00941-3.

[2] Liu J.; Wang G.; Song Z.; He X.; Luo K.; Ye Q.; Liao C.; Mei J. $FAPb_{1-x}Sn_xI_3$ Mixed Metal Halide Perovskites with Improved Light Harvesting and Stability for Efficient Planar Heterojunction Solar Cells. *Journal of Materials Chemistry* A **2017**, 5 (19), 9097-9106, https://doi.org/10.1039/C6TA11181E.

[3] Lian X.; Chen J.; Zhang Y.; Qin M.; Li J.; Tian S.; Yang W.; Lu X.; Wu G.; Chen H. Highly Efficient Sn/Pb Binary Perovskite Solar Cell via Precursor Engineering: A Two-Step Fabrication Process. *Advanced Functional Materials* **2019**, 29 (5), 1807024, https://doi.org/10.1002/adfm.201807024.

[4] Shao S.; Cui Y.; Duim H.; Qiu X.; Dong J.; Brink G. H. T.; Portale G.; Loi M. A. Enhancing the Performance of the Half Tin and Half Lead Perovskite Solar Cells by Suppression of the Bulk and Interfacial Charge Recombination. *Advanced Materials* **2018**, 30 (35), 1803703, https://doi.org/10.1002/adma.201803703.

[5] Eperon G. E.; Leijtens T.; Bush K. A.; Prasanna R.; Green T.; Wang J. T. W.; McMeekin D. P. et al. Perovskite-Perovskite Tandem Photovoltaics with Optimized Band Gaps. *Science* **2016**, 354 (6314), 861-865, https://doi.org/10.1126/science.aaf9717.

[6] Xi J.; Duim H.; Pitaro M.; Gahlot K.; Dong J.; Portale G.; Loi M. A. Scalable, Template Driven Formation of Highly Crystalline Lead-Tin Halide Perovskite Films. *Advanced Functional Materials* **2021**, 31 (46), 2105734, https://doi.org/10.1002/adfm.202105734.

[7] Park C.; Choi J.; Min J.; Cho K. Suppression of Oxidative Degradation of Tin–Lead Hybrid Organometal Halide Perovskite Solar Cells by Ag Doping. *ACS Energy Letters* **2020**, 5 (10), 3285-3294, https://doi.org/10.1021/acsenergylett.0c01648.
